# Prolonged Parenteral Nutrition Increases the Risk of Comorbidities in Very-Low-Birth-Weight Infants: A Prospective National Cohort Study in South Korea

**DOI:** 10.3390/nu17060996

**Published:** 2025-03-12

**Authors:** Seong Wan Kim, Yoong-A Suh, Seoheui Choi, Moon Sung Park, Jang Hoon Lee

**Affiliations:** Department of Pediatrics, Ajou University Hospital, Suwon 16499, Republic of Korea; kswan91@aumc.ac.kr (S.W.K.); mary5531@aumc.ac.kr (Y.-A.S.); shchoi@aumc.ac.kr (S.C.); drparkms@aumc.ac.kr (M.S.P.)

**Keywords:** very-low-birth-weight infants, parenteral nutrition, periventricular leukomalacia, bronchopulmonary dysplasia, retinopathy of prematurity, Korean neonatal network

## Abstract

**Background/Objectives**: There has been an increase in the incidence of comorbidities among very-low-birth-weight infants (VLBWIs), including periventricular leukomalacia (PVL), bronchopulmonary dysplasia (BPD), and retinopathy of prematurity (ROP). Parenteral nutrition is essential for very-low-birth-weight infants (VLBWIs) who are born with a birth weight of less than 1500 g, but a longer duration of parenteral nutrition is known to have a risk of comorbidity, such as ROP. This study aims to investigate the relationship between the duration of parenteral nutrition and the comorbidities of the VLBWIs. **Methods**: Using the prospective cohort of Korean neonatal network, we analyzed the perinatal and postnatal data before discharge of the total 2490 subjects born in 2021 and 2022. The primary outcomes were the diagnoses of PVL, BPD, and ROP. The secondary outcomes were the severity of BPD and ROP, treatment of ROP, and proposing the predictive model of comorbidities using the duration of parenteral nutrition. **Results**: This study found that prolonged parenteral nutrition exceeding 28 days was associated with a higher risk of PVL (odds ratio [OR] 1.71, 95% confidence interval [CI] [1.11, 2.64], *p* = 0.002) and BPD (OR 1.51, 95% CI [1.10, 2.08], *p* = 0.011). Furthermore, an intermediate duration of parenteral nutrition was found to be significantly associated with an increased risk of ROP in male subjects. Additionally, a prolonged duration of parenteral nutrition was observed to be linked to greater severity of BPD. Predictive models incorporating the duration of parenteral nutrition demonstrated a high degree of explanatory power in relation to both BPD and ROP. **Conclusions**: Longer duration of parenteral nutrition has a risk of critical comorbidities in VLBWIs. The nutrition strategy for shorter parenteral nutrition should be encouraged for the prevention of comorbidities.

## 1. Introduction

Advancements in the field of neonatology have led to substantial improvements in the survival rate of very-low-birth-weight infants (VLBWIs) born with a birth weight of less than 1500 g. However, this progress has been accompanied by an increased incidence of critical comorbidities, including periventricular leukomalacia (PVL), bronchopulmonary dysplasia (BPD), and retinopathy of prematurity (ROP) [1,2]. In South Korea, the survival rate of very-low-birth-weight infants (VLBWIs) increased from 86% to 88% when comparing the periods 2015–2016 and 2019–2020. Conversely, the incidence of BPD exhibited an upward trend, increasing from 29% to 33%. The incidence of ROP requiring therapy and PVL remained stable at 27% and 7%, respectively, indicating no improvement [2]. Previous studies have reported that various nutritional strategies in VLBWIs are effective in the prevention of these comorbidities. An adequate nutrient composition and calories in the early postnatal period have been demonstrated to be associated with a reduced risk of BPD and ROP [3,4,5,6]. The supplementation of vitamin A has been shown to decrease the likelihood of BPD [3]. In addition, the practice of breastfeeding has also been shown to contribute to the prevention of BPD and ROP [3,4,6].

Parenteral nutrition plays a pivotal role in the nutrition of VLBWIs, as enteral nutrition is limited immediately after birth. However, an increased duration of parenteral nutrition has been shown to be associated with an elevated risk of complications, including sepsis and cholestasis [7,8]. It is also known that the risk and severity of ROP increases with the duration of parenteral nutrition [5,9,10]. The association between PVL, BPD and duration of parenteral nutrition is not yet clearly understood, and the identification of this association would facilitate the planning of nutritional strategies for VLBWIs.

The objective of this study was to examine the relationship between the duration of parenteral nutrition and comorbidities. It was hypothesized that the risk of PVL and BPD would increase with a longer duration of parenteral nutrition. Additionally, this study sought to ascertain whether the duration of parenteral nutrition is associated with an increased risk of ROP, in line with the findings of previous studies.

## 2. Materials and Methods

### 2.1. Study Design and Data Source

This study was designed as a prospective cohort study with data from the Korean neonatal network (KNN). The KNN was initiated in 2013 and currently involves more than 70 neonatal intensive care units (NICUs), including the majority of tertiary NICUs in South Korea. The KNN cohort consists of very-low-birth-weight infants (VLBWIs) admitted to NICUs participating in the KNN. A comprehensive data set is collected, encompassing perinatal information, complications and treatment during hospitalization, and growth and development information from discharge to three years of age. The information is de-identified and stored to protect privacy.

### 2.2. Study Population

**Figure 1:**
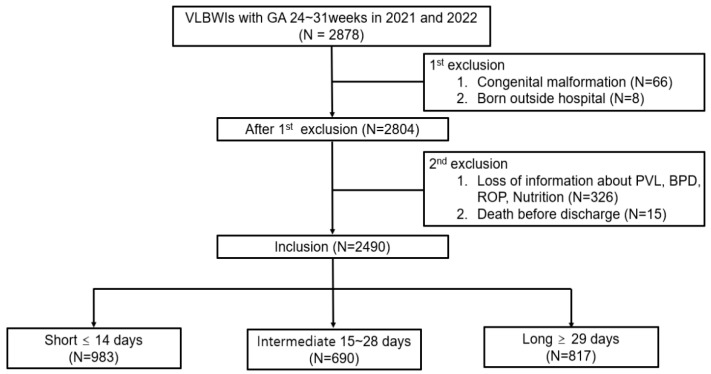
Flowchart of study population.

This study included VLBWIs born in 2021 and 2022 between 24 and 31 weeks of gestational age (GA) who were registered in the KNN. The perinatal and postnatal information of the subjects prior to discharge from the NICU was utilized. Infants with major congenital diseases and those born out of hospital were excluded from the study. Of the remaining subjects, those with a lack of information on PVL due to no cranial sonography or brain MRI during hospitalization, missing information on BPD due to discharge before 36 weeks of postmenstrual age (PMA), no assessment of ROP by ophthalmologist, or without data about nutrition were excluded from this study. The cases excluded tended to have a higher gestational age and birth weight, and examinations for comorbidities may not have been performed due to lower risk, but, in some cases, the information was simply not entered. Infants who died before discharge and could not be assessed for comorbidities were also excluded from this study. Following the application of these exclusion criteria, the total number of study subjects was 2490. Due to the wide distribution of parenteral nutrition duration of subjects, the subjects were categorized into three groups for the purpose of conducting a statistically robust analysis, referring to previous studies that classified subjects based on 14 or 28 days [11,12]. The categorization of subjects was as follows: ‘Short’ for 14 days or less, ‘Intermediate’ for 15 to 28 days, and ‘Long’ for 29 days or more. The respective numbers of subjects were 983, 690, and 817 (Figure 1).

### 2.3. Data Collection

Antenatal and birth information, as well as postnatal information prior to discharge from the NICU, was collected. The antenatal information comprised maternal hypertension, diabetes, antenatal steroid and antibiotics, chorioamnionitis, and premature rupture of membrane (PROM). Birth information included sex, multiple birth, mode of delivery, gestational age (GA), birth weight, 1 and 5 min APGAR score, and chest compression at birth. Postnatal information encompassed the presence of air leak syndrome, pulmonary hemorrhage, respiratory distress syndrome (RDS), patent ductus arteriosus (PDA) treatment, intraventricular hemorrhage (IVH) severity (Mild: Stages 1, 2; Severe: Stages 3, 4), necrotizing enterocolitis (NEC) (Stages 2 and above), sepsis proven with blood culture, and exposure to red blood cell (RBC) transfusion. We also collected data on PVL, BPD and ROP. PVL was defined and confirmed by cranial sonography or brain MRI during hospitalization in NICU. The diagnosis and severity classification of BPD were determined according to the 2001 NICHD guideline, while the stage of ROP was defined based on the maximum stage recorded in the ophthalmologist’s evaluation during hospitalization. The treatment status of ROP was also limited to interventions performed during hospitalization. We also utilized data on the duration of parenteral nutrition; however, detailed information on its composition and amount was not available in the KNN cohort.

### 2.4. Outcomes

The primary outcome of this study was the risk of diagnosis as PVL, BPD, and ROP prior to discharge. As secondary outcomes, the severity of BPD, the severity of ROP categorized into two groups according to the highest stage of ROP (Mild: Stage 1, 2. Severe: Stage 3 and above), and the treatment status of ROP were also analyzed. Furthermore, the identification of the predictive models and regression equations for PVL, BPD, and ROP using the variables in our study was an objective.

### 2.5. Statistical Analysis

Among the confounding variables, missing values were observed for chorioamnionitis, antenatal steroid, PROM, antenatal antibiotics, chest compression at birth, and 1 and 5 min APGAR scores. The frequency of missing values was not high, and they were not correlated with each other, thus indicating that they were MCAR (Missing Completely At Random). Consequently, the missing values were replaced with the mode value for categorical variables and the median value for continuous variables. Categorical variables are expressed as frequency and percentage and compared using the Pearson chi-squared test. Continuous variables are expressed as the mean with standard deviation and compared using ANOVA. Prior to conducting a binomial logistic regression analysis of primary and secondary outcomes, an investigation into the presence of multicollinearity was conducted. Initially, the R-value for each confounder was calculated to identify combinations of confounders with an R-value greater than or equal to 0.7. This was observed in the 1 and 5 min APGAR scores, and we proceeded to analyze the 5 min APGAR score. Subsequent to this, it was ascertained that the VIF values of the confounders were lower than 5, thereby confirming the absence of multicollinearity. An attempt was made to compare the outcomes for three groups based on the duration of parenteral nutrition received by subjects. However, in general, lower GA and birth weight may be linked to longer parenteral nutrition. To correct for this effect, generalized propensity score weighting was employed. For each group, the generalized propensity score and weights were calculated and corrected for GA and birth weight. The standardized mean difference was then checked to ensure that the three groups were well balanced, with a value of less than 0.1 to 0.15. The outcomes were then compared by duration of parenteral nutrition, and differences by gender were also compared. Predictive models about comorbidities were analyzed using binary logistic regression, and predictive power was assessed using the receiver operating characteristic (ROC) curves and the area under the curve (AUC) values. A *p*-value of less than 0.05 was defined as statistically significant. The R version 4.4.0 software was utilized for the execution of these analyses.

## 3. Results

### 3.1. Characteristics of Cohort and Their Comorbidities

The mean gestational age of the subjects in this study was 28.1 ± 2.0 days, and the mean birth weight was 1080 ± 257 g. A positive correlation was identified between longer PN duration and lower GA and birth weight, with statistically significant differences being identified. The proportion of subjects in this study who were female was 48.7%, with no statistical difference between the groups, but there was a higher proportion of females in the shorter duration. As previously mentioned, the duration of parenteral nutrition for the subjects in this study was distributed over a wide range, with a minimum of 0 days and a maximum of 209 days. The median duration was found to be 19 days and 10 to 34 days in the first and third quarters, respectively. The mean duration was found to be 25.61 days. In total, 161 infants had PVL, and BPD was reported in 1683 infants. As regards the patients with BPD, 745 patients had mild BPD, 280 patients had moderate BPD, and 658 patients had severe BPD. Furthermore, 904 infants were reported to have ROP, with 608 patients exhibiting mild severity (Stage 1: 307, Stage 2: 301) and 296 patients displaying severe ROP. However, patients with stage 4 or 5 were seldom reported (Stage 3: 290, Stage 4: 6, Stage 5: 0).

### 3.2. Comparison of Confounder by Duration of Parenteral Nutrition

In the context of perinatal variables, the duration of parenteral nutrition has been demonstrated to be associated with a number of factors, including maternal hypertension and diabetes, antenatal antibiotics, multiple births, gestational age (GA), group by birth weight, five-minute APGAR score, and compression at birth. Conversely, postnatal variables have been found to exhibit significant differences, with a higher incidence of complications observed in cases where parenteral nutrition is administered for a prolonged duration (Table 1).

### 3.3. Duration of Parenteral Nutrition and the Risk of PVL

In VLBWIs, the risk of PVL was found to increase significantly (odds ratio [OR] 1.71, 95% confidence interval [CI] [1.11, 2.64], *p*-value 0.002) in cases of prolonged parenteral nutrition (PPN) lasting more than 28 days, even after adjustment for gestational age (GA) and birth weight. However, the intermediate duration did not increase the risk of PVL. Among the other variables, the severity of IVH significantly elevated the risk, while a higher 5 min APGAR score and multiple births decreased the risk of PVL (Table 2). When the data were analyzed according to sex, a longer duration of PN was associated with a higher risk of PVL in males than in total (OR 2.31, 95% CI [1.33, 4.02], *p*-value 0.003), whereas in females, the PN duration was not associated with PVL. The duration of the parenteral nutrition was incorporated into the prediction model, and it was observed that an extended duration was associated with an increased likelihood of PVL. The model had an Akaike information criterion (AIC) of 1058.6 and a good explanatory power of 0.741 (Theorem 1 and Supplementary Figure S1).

log(P/1 − P)= −1.7045 + 0.6427⋅(Long PN) − 0.1090⋅(Intermediate PN) − 0.3165⋅(Antenatal antibiotics) − 0.1419⋅(5 min APGAR score) + 0.0006⋅(Birth weight(gm)) + 1.7490⋅(Severe IVH) + 0.4654⋅(Mild IVH)(1)

**Theorem** **1.** *Logistic regression model for parenteral nutrition and PVL; P: Probability of PVL occurrence; PN: Parenteral nutrition, APGAR: Appearance, Pulse, Grimace, Activity, Respiration, IVH: Intraventricular hemorrhage*.

### 3.4. Duration of Parenteral Nutrition and the Risk of BPD

The present study found that the prolonged duration of parenteral nutrition was associated with an elevated risk of BPD (OR 1.51, 95% CI [1.10, 2.08], *p*-value = 0.011). Conversely, an intermediate duration was associated with a trend towards an increased risk of BPD, though this association was not statistically significant. Being female, a history of antenatal antibiotics, and higher 5 min APGAR score were shown to decrease the risk of BPD. However, a history of RDS, PDA treatment and sepsis were associated with an increased risk of BPD, and RBC transfusion and a history of pulmonary hemorrhage significantly elevated the risk (Table 3). In male subjects, an increased duration was observed to be associated with an elevated risk of developing BPD, although this association was only statistically significant within the intermediate group (OR 1.46, 95% CI [1.01, 2.12], *p*-value 0.046). Conversely, in female subjects, a significant increase in the risk of BPD was observed among individuals in the prolonged group (OR 1.74, 95% CI [1.11, 2.73], *p*-value 0.017). In the predictive model of BPD, both long and intermediate durations of parenteral nutrition were included and associated with increasing the probability of BPD. Our model had an excellent explanatory power with 0.906 and AIC with 1812.6 (Theorem 2 and Supplementary Figure S1). In patients diagnosed with BPD, a longer duration exhibited a trend of increased the severity of BPD, though this was not statistically significant after adjusting for GA and birth weight (Supplementary Table S1). A similar trend was shown in the analysis of male subjects, but prolonged duration over 28 days mildly elevated the risk of BPD in female subjects (OR 1.15, 95% CI [1.01, 1.32], *p*-value 0.035).

log(P/1 − P) = 19.9832 + 0.7856⋅(Long PN) + 0.0791⋅(Intermediate PN) − 0.3679⋅(Sex) + 0.2175⋅(Maternal DM) − 0.1978⋅(PROM) − 0.3892⋅(Antenatal antibiotics) − 0.6075⋅(GA) − 0.1683⋅(5 min APGAR score) − 0.0011⋅(Birth weight (gm)) + 0.6521⋅(RDS) + 1.5008⋅(Pulmonary hemorrhage) + 0.4156⋅(PDA treatment) + 0.4367⋅(Sepsis) +0.9350⋅(NEC) +1.2712⋅(RBC transfusion) (2)

**Theorem** **2.** *Logistic regression model for parenteral nutrition and BPD; P: Probability of BPD occurrence; PN: Parenteral nutrition; DM: Diabetes mellitus; PROM: Premature rupture of membrane; GA: Gestational age; RDS: Respiratory distress syndrome; PDA: Patent ductus arteriosus; NEC: Necrotizing enterocolitis*.

### 3.5. Duration of Parenteral Nutrition and the Risk of ROP

The duration of parenteral nutrition was found not to be associated with the risk of ROP. Chorioamnionitis, chest compression at birth, history of RDS, PDA treatment, severe IVH, sepsis, and exposure to RBC transfusion were found to increase the risk of ROP. Conversely, a history of delivery by caesarean section has been shown to decrease the risk of ROP (Table 4). Interestingly, in male subjects, prolonged parenteral nutrition was associated with a reduced risk of ROP (OR 0.68, 95% CI 0.49, 0.94), *p*-value 0.020), but intermediate duration was associated with an elevated risk of ROP (OR 1.45, 95% CI [1.12, 1.88], *p*-value 0.005). However, no such association was observed in female subjects.

In the predictive model of ROP, both long and intermediate durations of parenteral nutrition were incorporated into the model and were found to be associated with a mild increase in the probability of ROP. The AIC of the model was 2393.1 with very good explanatory power of 0.831 (Theorem 3 and Supplementary Figure S1).

In patients diagnosed with ROP, no association was found between the duration of parenteral nutrition and ROP severity (Supplementary Table S2). Intermediate duration was associated with higher severity of ROP in male subjects (OR 1.70, 95% CI [1.05, 2.74], *p*-value 0.030), but prolonged duration was related to lower severity in female subjects (OR 0.53, 95% CI [0.30, 0.93], *p*-value 0.027). When analyzing the treatment of ROP, the risk of treatment was found to be lower in the prolonged duration (OR 0.59, 95% CI [0.40, 0.86], *p*-value 0.006) (Supplementary Table S3). No association was observed in either the male or female analyses.

## 4. Discussion

This study has found that prolonged parenteral nutrition in VLBWI was associated with an elevated risk of PVL and BPD, even after adjusting for GA, birth weight and other perinatal confounders. Furthermore, predictive models of comorbidities were presented, which demonstrated a satisfactory degree of predictive power. To the best of the authors’ knowledge, this is the first study to report an association between PVL and the duration of parenteral nutrition. Previous studies have shown that the duration of parenteral nutrition increases the risk of sepsis [8], which is known to be as a risk factor for PVL [13,14]. In addition, one study has reported an association between full enteral nutrition timing and long-term neurological development in preterm infants [15]. As the risk of prolonged parenteral nutrition increases with delayed full enteral timing, it is conceivable that the duration of parenteral nutrition may influence neurodevelopment and be associated with PVL. Furthermore, the impact of duration was observed to be more pronounced in male subjects. In the meta-analysis of risk factors for PVL [16], although the certainty of evidence was very low, male gender itself was reported to increase the risk of PVL and may be associated with it. When other risk factors associated with PVL were examined, it was found that IVH was the most significant risk factor, a finding that is consistent with the results of previous studies [16].

log(P/1 − P) = 12.7693 + 0.1860⋅(Long PN) + 0.1561⋅(Intermediate PN) + 0.4172⋅(Multiple birth) − 0.2038⋅(Maternal DM) + 0.3151⋅(Chorioamnionitis) − 0.2534⋅(PROM) + 0.5694⋅(Chest compression) + 0.2335⋅(RDS) + 0.2179⋅(PDA treatment) + 0.4919⋅(Severe IVH) + 0.0969⋅(Mild IVH) − 0.4011⋅(GA) − 0.0023⋅(Birth weight)(3)

**Theorem** **3.** *Logistic regression model for parenteral nutrition and ROP; P: Probability of ROP occurrence; PN: Parenteral nutrition; DM: Diabetes mellitus; PROM: Premature rupture of membrane; RDS: Respiratory distress syndrome; PDA: Patent ductus arteriosus; IVH: Intraventricular hemorrhage; GA: Gestational age*.

We found that the duration of parenteral nutrition was associated with the risk of BPD in both male and female subjects, although the odds ratios were not high. One study investigating a predictive model of BPD revealed that the risk of BPD increased with a longer duration of parenteral nutrition [17]. In addition, another study reported an increased prevalence of BPD in preterm infants with late full enteral timing [15]. Furthermore, our study demonstrated that other clinical factors, including pulmonary hemorrhage and RBC transfusion, were significantly associated with BPD. Pan et al. reported no significant association between pulmonary hemorrhage and BPD [18]. But their research included a smaller number of subjects than our study. Consequently, further studies are required in order to conduct a more in-depth investigation of this relationship. Additionally, it was observed that red blood cell (RBC) transfusion increased the incidence of BPD and severity, a finding that is in accordance with the results of other studies [19]. Furthermore, a predictive model of BPD was proposed, incorporating all of the aforementioned factors. It is anticipated that this model will be clinically useful given its high predictive power.

Contrary to the findings of studies conducted previously [5,9], no association was identified between the duration of parenteral nutrition and the risk of ROP following adjustment for GA and birth weight. Instead, an intermediate duration exhibited a tendency to augment the risk or severity of ROP more than a prolonged duration. This may be attributed to the observation that the ROP patients in our study exhibited a tendency towards lower severity. Indeed, the number of patients with Stages 4 and 5 in this study was seldom. Another possibility is that the subjects with long parenteral nutrition exhibited a higher proportion of infants with lower GA and birth weight compared to the other groups. These infants may have been subjected to earlier screening and intervention, potentially avoiding the necessity for treatment. Among other perinatal variables, chorioamnionitis was identified as a risk factor for ROP and was incorporated into a predictive model for ROP. The relationship between chorioamnionitis and ROP remains controversial in previous studies [20,21]. However, systemic inflammation induced by chorioamnionitis has the potential to influence retinal vascularization and the progression of ROP, as indicated by the results of the present study. Cesarean section was associated with a lower risk in our study, but the relationship between the mode of delivery and ROP remains controversial [22,23,24]. Further research on this relationship is needed in the future. RBC transfusion was found to be a significant risk factor for ROP [5], as with BPD. Consequently, measures aimed at minimizing exposure to RBC transfusions may also help to reduce the risk of comorbidities, similar to shortening the duration of parenteral nutrition.

Parenteral nutrition is essential for nutrition in the early postnatal phase in VLBWIs due to transient feeding intolerance. However, the findings of this study indicate that prolonged parenteral nutrition may also be associated with the risk. This can be attributed to the hypothesis that prolonged parenteral nutrition does not adequately raise serum Insulin-like Growth Factor-1 (IGF-1) levels, thereby increasing the likelihood of comorbidities. IGF-1 plays a crucial role in the processes of growth and organ development in the fetus [25]. Following preterm birth, there is a rapid decrease in serum IGF-1 levels as the maternal placental supply of IGF-1 disappears; in contrast to full-term infants, there is a gradual increase in serum IGF-1 levels [25,26,27]. However, IGF-1 performs an important role in preterm infants. In the brain, it regulates the maturation of cerebral blood vessels [28] and has a neuroprotective effect [29,30]. Furthermore, the postnatal serum IGF-1 level has been demonstrated to be associated with brain volume at term age [31,32]. In the lung, IGF-1 has been shown to play a role in alveolar development and maturation [25,33] and has an antioxidant and anti-inflammatory effect [34], which may contribute to the prevention of BPD. Studies have reported that the low serum IGF-1 levels immediately after birth were associated with an increased risk of BPD [35], and that delayed recovery of IGF-1 levels were associated with increased severity of BPD [34]. In the RCT for IGF-1 treatment for the prevention of ROP, the incidence of severe BPD was reduced by 53% in the IGF-1 group [36]. Consequently, an RCT is currently underway to ascertain the effectiveness of IGF-1 in the prevention of BPD (NCT03253263). IGF-1 also plays a crucial role in the pathogenesis of ROP as it regulates VEGF signaling, which is essential for the development of retinal blood vessels [37,38].

A number of studies have reported on the relationship between parenteral nutrition and postnatal serum IGF-1 levels. Engström et al. reported that the amount of enteral protein intake was found to be associated with changes in serum IGF-1 levels, and that a shorter duration of enteral protein intake reaching 3.5 g/kg was related to higher IGF-1 level at 30–33 weeks of PMA [39]. Yumani et al. reported that a higher calorie intake through parenteral nutrition from 8 to 12 days of age was associated with significantly lower IGF-1 levels at 2 weeks of age [40]. In a prospective study to identify clinical factors associated with serum IGF-1 level in the early postnatal period and at 35 weeks of PMA, a longer duration of parenteral nutrition was related to a lower IGF-1 level at 35 weeks of PMA [41]. These findings suggest that prolonged parenteral nutrition may result in a slower recovery of the serum IGF-1 level, potentially leading to delayed maturation and protection of vital organs, thereby increasing the risk of PVL, BPD, and ROP. Therefore, a strategy to shorten the duration of parenteral nutrition through active enteral feeding can be helpful to prevent these comorbidities.

This study has several limitations. Firstly, the NICUs in the cohort may have different policies for the treatment and nutrition of preterm infants, which can have introduced bias. Secondly, the cohort did not include much detailed information on nutrition in relation to comorbidities, such as composition and amount of parenteral nutrition, and type of lipid emulsion in the early postnatal period. In addition, the status for small for gestational age (SGA) is a significant confounding factor, but information regarding SGA was also missing from the data. Furthermore, the cohort utilized the 2001 NICHD criteria for the BPD, which does not reflect the recently adopted Jansen criteria. Despite these limitations, our study has several strengths and implications. Firstly, we utilized a prospective cohort that encompassed nearly all validated tertiary NICU centers in Korea. This approach enabled the inclusion of a substantial number of subjects and a comprehensive range of information from the antenatal to the postnatal period. Secondly, we employed a statistical strategy to adjust GA and birth weight, which are known influential confounders in studies related to VLBWIs. Notably, this study identified a novel relationship between the duration of parenteral nutrition and PVL, a significant comorbidity of VLBWIs. Finally, a highly accurate predictive model for BPD and ROP using the duration of parenteral nutrition was proposed, which can be clinically helpful. However, in order to overcome the aforementioned limitations and to determine the exact relationship between the duration of parenteral nutrition and IGF-1, a prospective study that includes both information on nutritional strategies and regular testing of IGF-1 levels and comorbidities from shortly after birth is needed.

## 5. Conclusions

The present study has identified a correlation between the administration of parenteral nutrition for a prolonged period and an elevated risk of major comorbidities in VLBWIs. Specifically, it was observed that parenteral nutrition that exceeded 28 days was associated with a heightened risk of both PVL and BPD. Furthermore, the findings of this study suggest that a longer duration of parenteral nutrition is a significant factor in the predictive model of major comorbidities. These findings may be influenced by the relatively lower serum IGF-1 levels that have been observed in conjunction with prolonged parenteral nutrition. Consequently, the optimization of nutritional strategies with the objective of minimizing the duration of parenteral nutrition may be a beneficial approach in reducing the risk of comorbidities in VLBWIs.

## Figures and Tables

**Table 1:** Demographic analysis about duration of parenteral nutrition and confounders.

Variable	Total (*N* = 2490)	Short (*N* = 983)	Intermediate (*N* = 690)	Long (*N* = 817)	*p*-Value
GA (week)	28.1 ± 2.0	29.2 ± 1.5	28.2 ± 1.8	26.7 ± 1.9	0.000 *
Birth weight (gm)	1080 ± 257	1222 ± 192	1085 ± 236	904 ± 235	0.000 *
Sex: Female	1212 (48.7%)	495 (50.4%)	343 (49.7%)	374 (45.8%)	0.125
Multiple birth	981 (39.4%)	434 (44.2%)	259 (37.5%)	288 (35.3%)	0.000 *
Maternal DM	424 (17.0%)	191 (19.4%)	104 (15.1%)	129(15.8%)	0.034 *
Maternal HTN	614 (24.7%)	247(25.1%)	189 (27.4%)	178 (21.8%)	0.038 *
Chorioamnionitis	714 (28.7%)	273 (27.8%)	184 (26.7%)	257 (31.5%)	0.089
PROM	904 (36.3%)	379 (38.6%)	231 (33.5%)	294 (36.0%)	0.102
Antenatal steroid	2299 (92.3%)	900 (91.6%)	638 (92.5%)	761 (93.1%)	0.446
Antenatal antibiotics	1683 (67.6%)	632 (64.3%)	452 (65.5%)	599 (73.3%)	0.000 *
Mode of delivery: C/Sec	2122 (85.2%)	840 (85.5%)	598 (86.7%)	684 (83.7%)	0.266
5 min APGAR score	7.2 ± 1.6	7.6 ± 1.5	7.1 ± 1.7	6.7 ± 1.7	0.000 *
Chest compression at birth	59 (2.4%)	10 (1.0%)	21 (3.0%)	28 (3.4%)	0.001 *
Pulmonary hemorrhage	59 (2.4%)	7 (0.7%)	12 (1.7%)	40 (4.9%)	0.000 *
Air leak syndrome	73 (2.9%)	14 (1.4%)	12 (1.7%)	47 (5.8%)	0.000 *
RDS	2061 (82.8%)	696 (70.8%)	595 (86.2%)	770 (94.2%)	0.000 *
IVH severity					0.000 *
Severe	115 (4.6%)	8 (0.8%)	29 (4.2%)	78 (9.5%)	
Mild	848 (34.1%)	245 (24.9%)	221 (32.0%)	382 (46.8%)	
No	1527 (61.3%)	730 (74.3%)	440 (63.8%)	357 (43.7%)	
PDA treatment	875 (35.1%)	162 (16.5%)	256 (37.1%)	457 (55.9%)	0.000 *
NEC	117 (4.7%)	2 (0.2%)	20 (2.9%)	95 (11.6%)	0.000 *
Sepsis	331 (13.3%)	37 (3.8%)	78 (11.3%)	216 (26.4%)	0.000 *
RBC transfusion	1513 (60.8%)	330 (33.6%)	452 (65.5%)	731 (89.5%)	0.000 *

* *p*-value < 0.05; DM: Diabetes mellitus; HTN; Hypertension; PROM: Premature rupture of membrane; C/Sec: Cesarean section; GA: Gestational age; RDS: Respiratory distress syndrome; PDA: Patent ductus arteriosus; NEC: Necrotizing enterocolitis.

**Table 2:** Statistical significance variables and their results about the risk of PVL.

Variable	OR	Lower.CI	Upper.CI	*p*-Value
Parenteral nutrition				
Long	1.71	1.11	2.64	0.002 *
Intermediate	0.89	0.63	1.25	0.505
Short (Reference)	-	-	-	-
IVH severity				
Severe	7.70	5.11	11.62	0.000 *
Mild	1.66	1.20	2.30	0.002 *
No (Reference)	-	-	-	-
Multiple birth	0.61	0.39	0.95	0.029 *
5 min APGAR score	0.81	0.71	0.92	0.002 *

* *p*-value < 0.05; IVH: Intraventricular hemorrhage.

**Table 3:** Statistical significance variables and their results about the risk of BPD.

Variable	OR	Lower.CI	Upper.CI	*p*-Value
Parenteral nutrition				
Long	1.51	1.10	2.08	0.011 *
Intermediate	1.13	0.88	1.44	0.342
Short (Reference)	-	-	-	-
Sex (Female)	0.70	0.54	0.91	0.007 *
Antenatal antibiotics	0.67	0.50	0.90	0.009 *
5 min APGAR score	0.79	0.71	0.88	0.000 *
RDS	2.33	1.72	3.17	0.000 *
Pulmonary hemorrhage	7.66	1.72	34.13	0.008 *
PDA treatment	2.62	1.91	3.58	0.000 *
Sepsis	2.89	1.67	4.99	0.000 *
RBC transfusion	5.12	3.91	6.70	0.000 *

* *p*-value < 0.05; RDS: Respiratory distress syndrome; PDA: Patent ductus arteriosus.

**Table 4:** Statistical significance variables and their results about the risk of ROP.

Variable	OR	Lower.CI	Upper.CI	*p*-Value
Parenteral nutrition				
Long	0.88	0.69	1.12	0.303
Intermediate	1.13	0.94	1.36	0.195
Short (Reference)	-	-	-	-
Chorioamnionitis	1.61	1.25	2.09	0.003 *
Mode of delivery: C/Sec	0.66	0.48	0.92	0.015 *
Chest compressionAt birth	2.03	1.04	3.96	0.039 *
RDS	1.53	1.07	2.19	0.021 *
PDA treatment	1.76	1.38	2.24	0.000 *
IVH severity				
Severe	1.88	1.20	2.93	0.006 *
Mild	1.22	0.90	1.64	0.196
No (Reference)	-	-	-	-
Sepsis	1.70	1.21	2.38	0.002 *
RBC transfusion	2.29	1.75	3.02	0.000 *

* *p*-value < 0.05; C/Sec: Cesarean section; RDS: Respiratory distress syndrome; PDA: Patent ductus arteriosus; IVH: Intraventricular hemorrhage.

## Data Availability

Data are contained within this article and the Supplementary Materials.

## Supplementary Materials

The following supporting information can be downloaded at: https://www.mdpi.com/article/10.3390/nu17060996/s1, Figure S1: ROC curve of predictive model of PVL, BPD, ROP; Table S1: Statistical significance variables and their results about the severity of BPD; Table S2: Statistical significance variables and their results about the severity of ROP; Table S3: Statistical significance variables and their results about the treatment of ROP.

## Abbreviations

The following abbreviations are used in this manuscript:

VLBWI	Very-Low-Birth-Weight Infant
PVL	Periventricular Leukomalacia
BPD	Bronchopulmonary Dysplasia
ROP	Retinopathy of Prematurity
IGF-1	Insulin-like Growth Factor 1
KNN	Korean Neonatal Network
NICU	Neonatal Intensive Care Unit
GA	Gestational Age
PMA	Postmenstrual Age
