# Supplementary material for: Prolonged Parenteral Nutrition Increases the Risk of Comorbidities in Very-Low-Birth-Weight Infants: A Prospective National Cohort Study in South Korea
Source: Nutrients. 2025 Mar 12;17(6):996. doi: 10.3390/nu17060996 (PMC11946462; doi:10.3390/nu17060996)
Supplement: Supplementary file 1 [file nutrients-17-00996-s001.zip › nutrients-3510456-supplementary.pdf]

< Supplemental Figure S1>

1. ROC curve of TPN duration & PVL

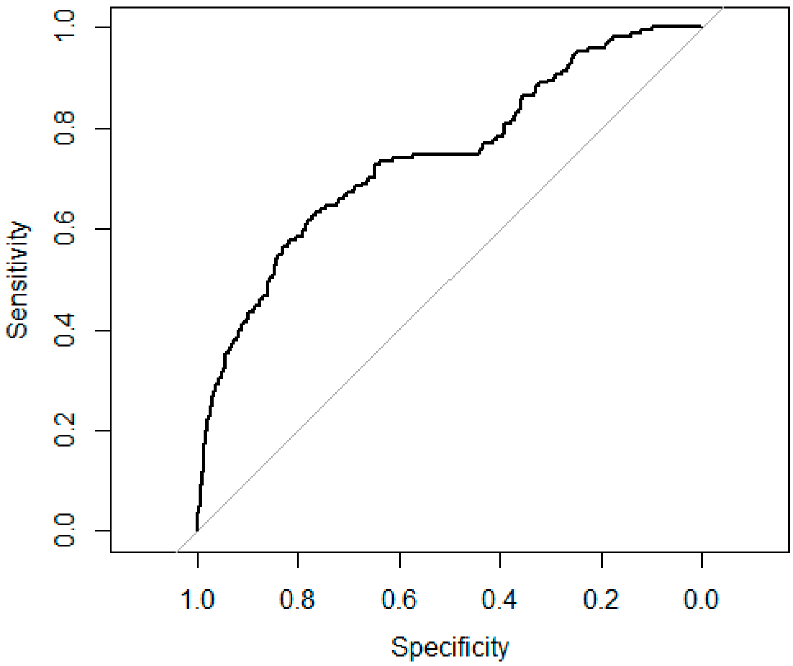

2. ROC curve of TPN duration & BPD

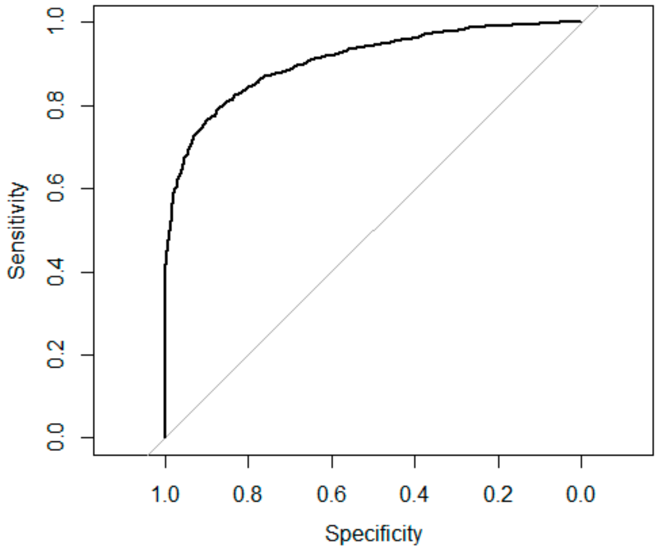

3. ROC curve of TPN duration & ROP

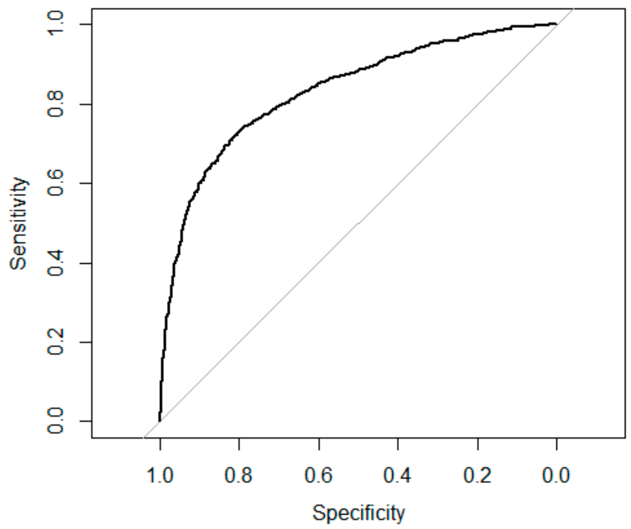

Table S1. Statistical significance variables and their results about the severity of BPD

Variable	OR	Lower.CI	Upper.CI	P-value
Parenteral nutrition				
Long	1.09	1.00	1.20	0.061
Intermediate	1.06	0.98	1.15	0.123
Short (Reference)	-	-	-	-
Antenatal antibiotics	0.89	0.80	0.99	0.026*
5-min APGAR score	0.96	0.94	0.99	0.021*
RDS	0.75	0.63	0.89	0.001*
Pulmonary hemorrhage	1.33	1.04	1.70	0.025*
Air leak syndrome	1.45	1.15	1.82	0.002*
PDA treatment	1.19	1.07	1.31	0.001*
Sepsis	1.25	1.10	1.42	0.001*
NEC	1.34	1.11	1.62	0.002*
RBC transfusion	1.72	1.54	1.92	0.000*

* P-value <0.05; RDS: Respiratory distress syndrome; PDA: Patent ductus arteriosus; NEC: Necrotizing enterocolitis

Table S2. Statistical significance variables and their results about the severity of ROP

Variable	OR	Lower.CI	Upper.CI	P-value
**Parenteral nutrition**				
**Long**	0.70	0.48	1.03	0.071
**Intermediate**	1.30	0.94	1.79	0.116
**Short (Reference)**	-	-	-	-
**5-min APGAR score**	0.91	0.81	1.01	0.076
**Pulmonary hemorrhage**	2.60	1.19	5.67	0.017*
**IVH severity**				
**Severe**	1.45	0.98	2.14	0.065
**Mild**	0.89	0.66	1.20	0.434
**No (Reference)**	-	-	-	-
**Sepsis**	1.45	0.98	2.15	0.064
**RBC transfusion**	3.25	1.66	6.38	0.001*

* P-value <0.05; IVH : Intraventricular hemorrhage

Table S3. Statistical significance variables and their results about the treatment of ROP

Variable	OR	Lower.CI	Upper.CI	P-value
Parenteral nutrition				
Long	0.59	0.40	0.86	0.006*
Intermediate	1.16	0.84	1.60	0.379
Short (Reference)	-	-	-	-
Chorioamnionitis	1.55	1.10	2.19	0.013*
5-min APGAR score	0.78	0.70	0.87	0.000*
Pulmonary hemorrhage	3.16	1.46	6.86	0.004*
PDA treatment	1.67	1.17	2.38	0.005*
IVH severity				
Severe	1.71	1.13	2.58	0.012*
Mild	1.04	0.76	1.42	0.797
No (Reference)	-	-	-	-
NEC	1.89	1.00	3.59	0.050
RBC transfusion	3.27	1.73	6.15	0.000*

* P-value <0.05; PDA: Patent ductus arteriosus; IVH: Intraventricular hemorrhage; NEC: Necrotizing enterocolitis
